# Adsorption of direct blue 106 dye using zinc oxide nanoparticles prepared via green synthesis technique

**DOI:** 10.1007/s11356-023-26954-x

**Published:** 2023-05-04

**Authors:** Ahmed Eleryan, Uyiosa O. Aigbe, Kingsley E. Ukhurebor, Robert B. Onyancha, Mohamed A. Hassaan, Marwa R. Elkatory, Safaa Ragab, Otolorin A. Osibote, Heri S. Kusuma, Ahmed El Nemr

**Affiliations:** 1 Environment Division, National Institute of Oceanography and Fisheries (NIOF), Kayet Bey, Elanfoushy, Alexandria Egypt; 2 Department of Mathematics and Physics, Faculty of Applied Sciences, Cape Peninsula University of Technology, Cape Town, South Africa; 3 Department of Physics, Faculty of Science, Edo State University Uzairue, Edo State, Nigeria; 4 Department of Technical and Applied Physics, School of Physics and Earth Sciences Technology, Technical University of Kenya, Nairobi, Kenya; 5 Polymer Materials Research Department, Advanced Technology and New Materials Research Institute, SRTA-City, New Borg El-Arab City 21934, Alexandria, Egypt; 6 Department of Chemical Engineering, Faculty of Industrial Technology, Universitas Pembangunan Nasionsal Veteran Yogyakarta, Sleman, Indonesia

**Keywords:** Direct blue 106 dye, Industrial wastes, Nanoparticles, Sorption, Water treatment

## Abstract

Zinc oxide nanoparticles (ZnO-NPs) have in recent times shown effective adsorption capability for the confiscation of colour contaminants from aqueous environments (aquatic ecosystems or water bodies) due to the fact that ZnO contains more functional groups. Direct blue 106 (DB106) dye was selected for this present study as a model composite due to its wide range of uses in textiles (cotton and wools), woods, and paper industries, as well as their therapeutic applications, along with its potential for impairments. This study therefore focuses on the use of DB106 dye as a model composite due to its wide range of uses in textiles (cotton and wools), woods, and paper industries, as well as their therapeutic applications and their potential for impairments. Furthermore, the surface functionalization, shape, and composite pore size were revealed by TEM, FTIR, UV, and BET techniques. The current study uses green synthesis method to prepare ZnO-NPs as an adsorbent for the DB106 dye molecules adsorption under various conditions using the batch adsorption process. The adsorption of DB106 dye to the ZnO-NPs biosorbent was detected to be pH-dependent, with optimal adsorption of DB106 (anionic) dye particles observed at pH 7. DB106 dye adsorption to the synthesized ZnO-NPs adsorbent was distinct by means of the linearized Langmuir (LNR) and pseudo-second-order (SO) models, with an estimated maximum adsorption capacity (*Q*_m_) of 370.37 mg/g.

**Graphical Abstract:**

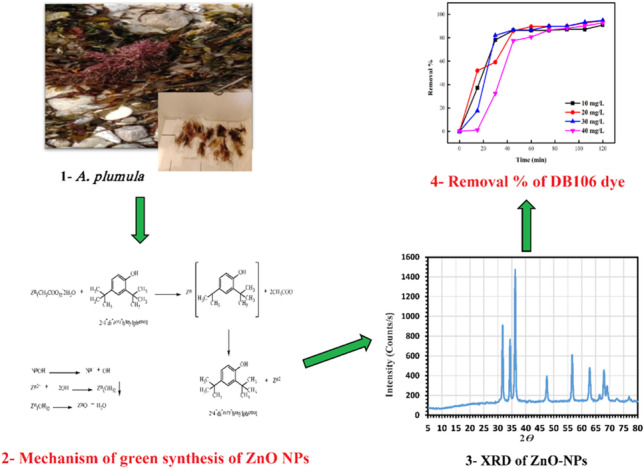

## Introduction

The issues of ecological destruction that are harming natural resources and causing a variety of environmental effluences (in the form of toxic wastes) as a result of increased industrial activity, a significant increase in population, and constant high-tech development are now a serious threat to the human being and its environment on a global scale (Onyancha et al. 2021a; Ukhurebor and Aidonojie 2021; Ukhurebor et al. 2021a). The majority of these environmental toxic wastes are the result of industrial processes, including dyes (Aigbe et al. 2021a; Eldeeb et al. 2022a, b), heavy metals (HMs) (El-Nemr et al. 2022; Aigbe et al. 2022; Onyancha et al. 2022; Eleryan et al. 2022), wastewater (Aigbe et al. 2021a, b; Sudarni et al. 2021), gas flares, and crude oil spills (Ukhurebor et al. 2021b; Onyancha et al. 2021; Ukhurebor et al. 2022a), among others. These environmental effluences rising from industrial operations are aggravating the negative environmental, health, and climatic challenges that are jeopardizing both the terrestrial, aquatic, and atmospheric ecosystems (Ukhurebor et al. 2022a; Ukhurebor et al. 2021c; Onyancha et al. 2021; Ukhurebor et al. 2022b).

The textile industries consume several quantities of chemicals and water and, in return, emit a lot of hazardous and non-biodegradable chemical effluents, such as dyes, and are a significant cause of industrial pollution (Jaafari et al. 2018; Ali et al. 2020a; Eldeeb et al. 2022a, b; Al-Arjan 2022). Due to dyes’ beneficial qualities, such as vivid colours, water resistance, and simplicity of applications (Al-Arjan 2022; Yang et al. 2022), they are frequently used in several textile-based businesses (Eldeeb et al. 2022a, b; Ali et al. 2020a; Hassaan et al. 2022). Synthetic dyes are used extensively in several industries, including textile dyeing (amounting to about 60%), paper (amounting to about 10%), and plastic products (amounting to about 10%) (Al-Arjan 2022).

As noted by several reports, there are about a hundred thousand commercially existing dyes with a manufacturing capability of over 7.0 × 10^5^ metric tons annually, with the industries involved in textile manufacturing using approximately ten thousand composites (Al-Arjan 2022). Industrial effluents from dyes, in particular, necessitate not only the management of difficult wastewater with high biological and chemical oxygen demands, deferred constituent parts, and dangerous chemicals, but also the treatment of dyes that are perceptible to human senses at very low concentrations (Al-Arjan 2022; Kusuma et al. 2023; Neolaka et al. 2023). When dyes are discharged into aquatic settings (water bodies), reductive azo linkage cleavage takes place, creating harmful amines that can harm key organs like the kidney, brain, and liver as well as the reproductive and central neurological systems (Al-Arjan 2022; Kusuma et al. 2023; Neolaka et al. 2023). Due to the existence of metals, chlorides, aromatics, and other impurities, synthetic dyes may also negatively affect some aquatic life’s ability to photosynthesise. As a result, it is crucial to remove them from aquatic ecosystems (water bodies), and this is the subject of various scientific investigations (Al-Arjan 2022; Bayramoglu and Arıca 2007; Hassaan et al. 2020a, b; El Nemr et al. 2021a; Hassaan et al. 2021).

Exchange of ions (Guida et al. 2022), electrolytic reduction (Golder et al. 2011), membrane and oxidation technologies (Schwermer et al. 2018; Deng and Zhao 2015), chemical precipitation (Harper and Kingham 1992), flocculation/coagulation (Song et al. 2004; Semerjian and Ayoub 2003), and adsorption by biological means (Sudarni et al. 2021a; El-Nemr et al. 2022), are some of the notable developed scientific investigations (methods) that have been used for the dyes and impurities adsorption from aquatic ecosystems (water bodies) and wastewaters. Due to its ease of use, high efficiency, and versatility for using a variety of adsorbents, adsorption by the biological process has recently become the most widely used processes for the adsorption of dyes from aquatic ecosystems and wastewaters (Zazouli et al. 2016; Azari et al. 2017, 2020; Al-Arjan 2022; El Nemr et al. 2021a; Hassaan et al. 2021; Hassaan et al. 2020a, b).

Diverse nanoparticles (NPs) have also been investigated for dye removal by biological processes because of the ease with which their surface functionality may be adjusted and their high surface-to-volume ratio for increased removal efficiency (Ghiloufi et al. 2016). It is believed that dyes from aqueous solutions (aquatic ecosystems) can be adsorbable by nanoscale metal oxides, such as aluminium, cerium, ferric, and magnesium oxides (Agrawal and Sahu 2006). Additionally, the effectiveness of these NPs as very effective adsorbents for the sorption of metal ions from aquatic ecosystems and wastewaters has been thoroughly studied. They have many advantages, containing unsaturated surfaces, ease of use, high ability, rapid kinetics, and favourable dye removal in aquatic ecosystems and wastewaters (Agrawal and Sahu 2006).

As a low-toxicity substance, zinc oxide (ZnO) has several applications, including those in the biomedicine (Azizi et al. 2016), catalysts (Zeng et al. 2008), gas sensing (Jing and Zhan 2008), and solar cells (Chou et al. 2007; Emegha et al. 2022). Additionally, ZnO is a member of the group of metal oxides that have a significant economic impact due to their impressive uses in a variety of industrial sectors, including solar cells, catalysis, paints, electronic devices, UV light-emitting devices, cosmetics, and biomedicine. Similar to ZnO-NPs, these semiconductors have drawn interest for their widespread variety of uses in optics, optoelectronics, electronics, and dye removal using environmentally friendly synthesis materials such as bacteria, fungi, and marine macroalgae (Al-Arjan 2022). ZnO has also been shown to be more efficient than other metals in the bio-synthesis of NPs for biomedical purposes (Naseer et al. 2020). The most significant metal oxide NPs are ZnO-NPs due to their special chemical and physical characteristics, which expand their usefulness (Elia et al. 2014). It can be difficult to remove certain contaminants from the environment; consequently, adsorption by biological processes or techniques is typically considered to be easier and more efficient. The useful application of ZnO in water purification, containing contamination removal and reuse, has drawn significant consideration recently due to its huge theoretical specific surface area (Yu et al. 2015). ZnO has demonstrated better adsorption performance because it contains more functional groups. ZnO may therefore be more useful in adsorption technologies (adsorption by biological processes or techniques). In addition, it was discovered that ZnO worked better as an adsorbent to remove sulphur compounds and H_2_S than other adsorbents such as activated carbon, phosphate, and iron oxide (Al-Arjan 2022; Hassan et al. 2008).

Recently, it was discovered that ZnO-NPs effectively absorbed colours from aquatic ecosystems or water bodies (aqueous environments) (Al-Arjan 2022; Wang et al. 2010). Direct blue 106 (DB106) dye was selected for this present study as a model composite due to its wide range of uses in textiles (cotton and wools), woods, and paper industries, as well as their therapeutic applications, along with its potential for impairments. In this study, the adsorption batch process was used to synthesize a composite of ZnO-NPs as an adsorbent for the adsorption of DB106 dye from aquatic ecosystems or water bodies (aqueous environments) under varied circumstances. The pH, starting concentration of DB106 dye, time of contact, dosages of adsorbent, and process temperature were the core factors that were assessed. Furthermore, the surface functionalization, shape, and the composite pore size were revealed by TEM, FTIR, UV, and BET techniques. The adsorption performance of the developed material was also examined using adsorption optimization analysis, kinetics, isotherms, and thermodynamics. Therefore, the aim of the current study was to reconsider and investigate the potential benefits of utilizing green synthesis method for ZnO-NPs preparation for DB106 dye removal. The purpose of this study was to demonstrate modern methods of removing DB 106 dye using more widely available, inexpensive, high-efficiency, and environmentally friendly NPs.

## Experimental

### Materials and equipment

**Fig. 1:**
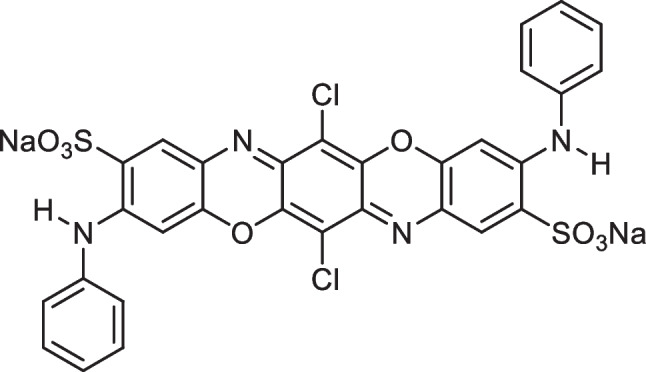
DB106 dye chemical formula (MF: C_30_H_16_C_l2_N_4_Na_2_O_8_S_2_) (MW: 741.49 g/mol) (C.I. 51,300) (Also named as: Fast Blue BL, Direct Brill Blue BL, Blue BL-0l, Sky Blue D-F2G) (CAS number: 6527–70-4)

NaOH, Zn(CH_3_CO_2_)_2_^.^2H_2_O, HCl, DB106 dye (Fig. 1), and EtOH were obtained from Sigma (Sigma–Aldrich, Germany). Red alga *A. plumula* (*Antithamnion plumula*) was obtained from Kiel, Germany. High-performance double beam spectrophotometer instrument model Pg/T80 UV/Visible matched with 1 cm glass cells as an optical path was used for dye concentration analysis. JSOS-500 shaker equipment and JENCO-6173 pH meter were used during this experimental work. The GC-mass spectrometry (Agilent 7890A) linked with a MS detector (Agilent 5975C) and HP5 column chromatography was applied for the chemical analysis of algae.

### Batch adsorption studies

Batch DB106 dye adsorption investigation was accompanied to examine the effects of several variables on the adsorption isotherm and reaction kinetics, including pH, starting concentration of dyes, ZnO-NPs doses, speed of agitation, time of contact, and process temperature. The necessary quantity of ZnO-NPs was added to dye solutions with known concentrations, and then the mixture was agitated for the definite times. Initial kinetic measurements were used to obtain an estimate of the time required to reach equilibrium conditions. After that, the solutions were filtered, and a UV–Vis spectrophotometer was used to monitor the absorbance at *λ*_max_ 660 nm to quantify the remaining concentration of DB106 dye in the filtrate. By regulating the pH of the dye solutions with NaOH and HCl solutions, the pH impact was also studied. Analysing the dye solution’s ability to bind to surfaces at various contact times allowed for the determination of the adsorption kinetics. Solutions of several concentrations were stirred with the ZnO-NPs until equilibrium was reached in order to examine adsorption isotherms. Using Eq. (1), it was possible to calculate the equilibrium adsorption capacity (*q*_e_).

1 $${q}_{e}=\frac{\left({C}_{0}-{C}_{t}\right)}{W}\times V$$

The capability of the ZnO-NPs to remove DB106 dye from water at an equilibrium time is known as the adsorption capacity (*q*_e_), which is represented as mg DB106 dye per g ZnO-NPs. In this equation, *C*_0_ (mg/L) stands for the DB106 dye’s beginning concentration, and *C*_t_ (mg/L) for the dye’s remaining concentration after the removal process has been finished for a predetermined amount of time (min). Use the following Eq. (2) to compute how much DB106 dye has been removed from water.

2 $$\%\;dye\;removal=\frac{\left(C_0-C_t\right)}{C_0}\times100$$

DB106 dye removal was investigated via mixing ZnO-NPs (0.2 g/L) and DB106 dye solution (50 mL) with a starting pH value ranging between 3 and 11. 0.1N HCl or 0.2N NaOH solutions were used to regulate the levels of pH, and the results showed that pH affected DB106 dye adsorption. The mixtures were agitated at 200 rpm for 120 min at 25 °C afore being sampled for DB106 dye measurement, which took place after 120 min.

In order to conduct the isotherm research, DB106 dye solutions (50 mL) was mixed at 200 rpm for 2 h at 25 °C with varied starting DB106 dye solution concentrations (10–40 mg/L) and varying doses of ZnO-NPs (40–200 mg) until the dye solutions attained equilibrium. By shaking 50 mL of starting DB106 dye concentration for ZnO-NPs with varying adsorbent dosages (40, 80, 160, and 200 mg) at various interval times at 25 °C for different periods at different intervals, the effect of ZnO-NPs dose and contact length on DB106 dye removal was investigated.

### Preparation of ZnO-NPs

For ZnO-NPs synthesis, 2.195 g of Zn(CH_3_CO_2_)_2_^.^2H_2_O was liquefied in 10 mL of red algae *A. plumula* extract and 90 mL distilled water (DW) in constant agitation at room temperature for 4 h. Then, a solution of 1.0 M NaOH was added drop-wise into the reaction mixture until the pH reached 10, and the agitation was continued for 3 h at 70 °C. The contaminants were removed by filtering and repeatedly washing the resultant white precipitate with DW and ethanol. The resultant white powder was then dried for 4 h at 80 °C in a vacuum oven (Hassaan et al. 2019; Albo Hay Allah and Alshamsi 2022; Hassaan et al. 2020a). The dried powder was then carefully collected and used for further research after being calcined for 3 h at 550 °C to produce pure, pale white ZnO-NPs (Hassan et al. 2015; Hassaan et al. 2019; Hassaan et al. 2020a; Amirante et al. 2018).

### Classification and analysis

ZnO-NPs sample was analysed using the subsequent instrument: Fourier transform infrared (FTIR) spectroscopy model VERTEX70 linked to V-100 platinum ATR model, Bruker, Germany, in the 400–4000 cm^−1^ wavenumber range. The Raman spectra (RS) model V-100 VERTEX70, Germany, the ZnO-NPs samples were excited to a laser beam of 532 nm (green laser) and the samples were exposed to the laser beam for 1 s at 10 mW power with aperture 25 × 1000 mm. XRD analysis was made by a Bruker Meas Srv (D2-208,219)/D2-2,082,019 diffractometer that controls at 30 kV, 10 mA using Cu tube (λ = 1.54 Å) in the range between 0 and 100°. The crystallite sizes were measured for ZnO-NPs by the method of Scherrer. The synthesized ZnO-NPs was categorized by a Transmission Electron Microscope (TEM) (JEOL, Model JSM 6360LA, Tokyo, Japan). Thermogravimetric (TGA) analysis of the ZnO was performed by (the TERIOS SDT650 instrument). The BELSORP—Mini II, BEL Japan, Inc. was used to measure the mean diameter of the pores and the BET (Brunauer–Emmett–Teller) specific surface area.

### Preparation of A. plumula extract

The harvested red algae *A. plumula* (obtained from Kiel, Germany) were placed in a polythene bag and delivered to the lab after being rinsed with water to remove sand and epiphytes. To further eliminate salts and other pollutants, the algae were washed with DW before being shade-dried and stored. Using a mortar and pestle and an electric blender, the dried algae were broken up into minute bits. 20 mL of methanol and 2 g of the powdered algae sample were combined to create the extract (Ragunathan et al. 2019). The mixture was then incubated in at 25 °C for the following day. After that, the extract was filtered with a funnel and No. 1 Whatman filter paper. In a conical flask of 100 mL, the extract was collected. The collected extract was evaporated under vacuum to collect the crude extract of the algae. Following collection, the extracted was used for GC–MS analysis.

### *Point of zero charge (pH*_*pzc*_*)*

The pHpzc of ZnO-NPs was assessed by the pH drift process (Shoaib et al. 2022a, b), in which suspensions of ZnO-NPs in 0.01 M NaCl solutions with various beginning pH values (3–11) were used for this investigation. The starting pH values (pH initial) were designed in contradiction of the end pH values (pH final), and the point of crossing between the pH_initial_ – pH_final_ curve and the pH_initial_ = pH_final_ line give the point of zero charge value.

## Results and discussion

### Red algae extract analysis using GC–MS

**Fig. 2:**
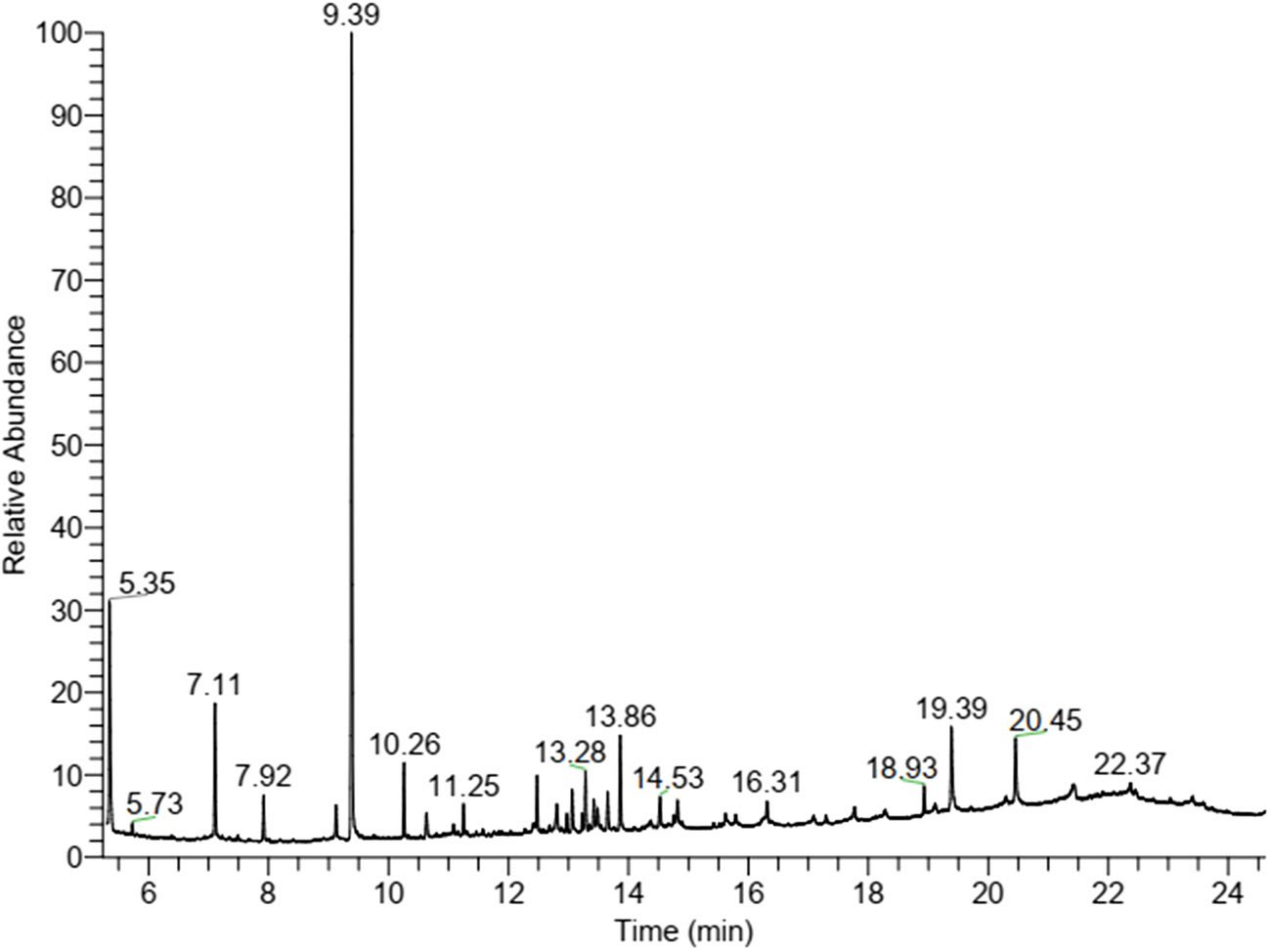
GC–MS analysis chromatogram of red algae *A. plumula* extract

**Scheme 1:**
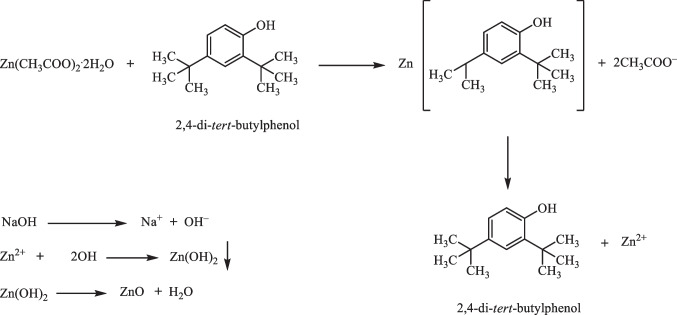
Preparation mechanism of green ZnO-NPs

Many compounds, such as alcohol, phenols, ethers, and esters were detected in the GC–MS chromatogram by application of NIST library. The algae *A. plumula* extract analysis by GC–MS showed eleven chemical compounds as major (Fig. 2). Between these compounds, at retention time (RT) 7.11 min is compound dodecamethylcyclohexasiloxane, RT 7.92 min is compound 5-octadecenal, RT 9.39 min is compound 2,4-bis (1,1-dimethylethyl) phenol, RT 10.26 min is compound 3-hydroxyspirost-8-en-11-one, RT 13.41 min is compound estriol 16-glucuronide, and RT 14.82 min is compound 9-desoxy-9x-chloroingol-3,7,8,12-tetraacetate were identified by NIST library. The following is a hypothesis on how these bioactive chemicals encourage bio-reduction: as indicated in Scheme 1, during the early stage, the metal ions go through the activation phase, where the development rate of particles is typically slow as the metal ions are reduced from their salt precursors by the achievement of biomolecule metabolites of plant with reduction abilities. In the present study, the reduction of zinc ions happens due to the presence of biomolecules in red algae *A. plumula* extract such as 2,4-bis(1,1-dimethylethyl) phenol.

### ZnO-NPs categorisation

#### FTIR investigation

**Fig. 3:**
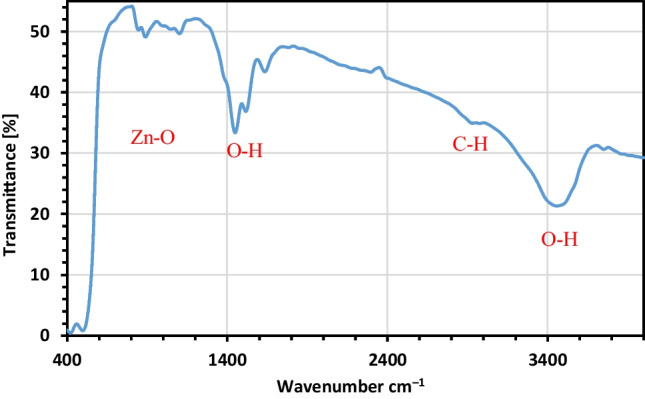
The FTIR curve of synthesized green ZnO-NPs

Phytoconstituents present in the red algae extract play a vital role in the reduction of ZnO-NPs and act as a covering agent on ZnO-NPs. FTIR analysis of synthesized ZnO-NPs is presented in Fig. 3 which verified in a wavenumber ranged between 400 and 4000 cm^−1^ to identify the structure of prepared green ZnO-NPs. The band at 503.43 cm^−1^ is matched to ZnO, confirming the formation of ZnO-NPs (Hassaan et al. 2020a, b). The peaks at 887.2, 1448.5, and 1641.4 cm^−1^ matched respectively to C–H, C–C, and H–O–H, and are corresponding to the presence of an organic compound. The broad band at 3462.4 cm^−1^ is matching to the OH, which characterises the existence of H_2_O molecules on the ZnO-NPs surface (Alim et al. 2005; Ashkenov et al. 2003; Xu et al. 2007; Ali et al. 2020b; Soliman et al. 2018; Salah et al. 2021).

#### Raman spectra analysis

**Fig. 4:**
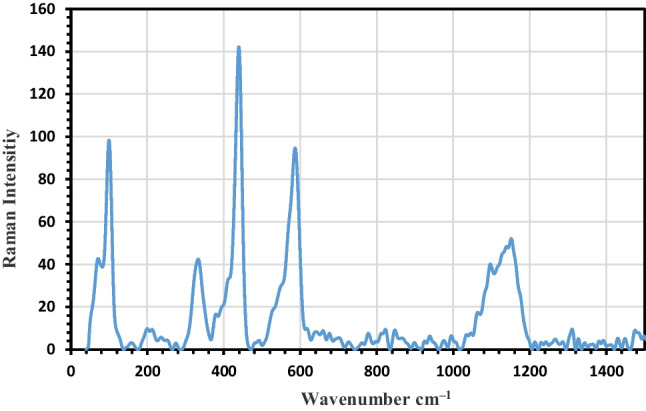
Raman analysis of synthesized green ZnO-NPs

In this approach, electrons are excited into higher energy levels as they relax back to a certain vibrational level, which offers information on the sample’s structural and vibrational features, which are supported by FTIR steps. The green synthesized ZnO-NPs are Raman active modes. In this study, ZnO-NPs show that the prominent vibration bands (secondary vibration) at 101.5, 337, 438, and 589.5 cm^−1^ match to the *E*2L, *E*2H–*E*2L, *E*2H, *E* 1 (LO) essential phonon modes of hexagonal ZnO-NPs, respectively (Fig. 4). The maximum band is 438 cm^−1^ corresponding to *E*2 mode wurtzite structured ZnO and a very sharp feature. The band at 589.5 cm^−1^, situated between A1 1 (LO) and E1 (LO) optical phonon mode, arises due to multi-phonon and resonance processes and is related to oxygen imperfection. Also, the acoustic combination of A1 and *E*2 is observed around 1155 cm^−1^.

#### XRD analysis

**Fig. 5:**
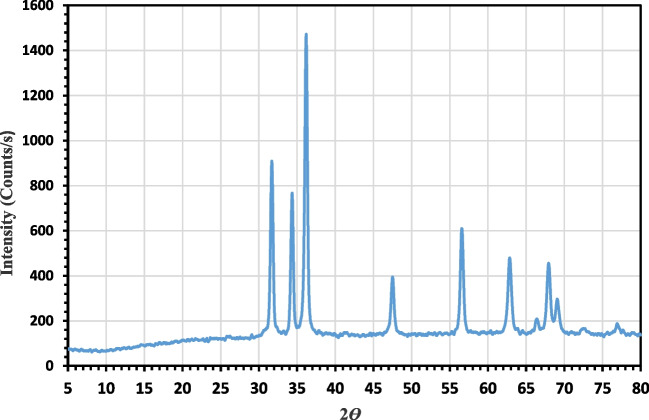
XRD spectra analysis of prepared green ZnO-NPs

Figure 5 depicts the synthesised green ZnO-NPs’ XRD diffraction pattern. The index numbers for the peaks 2 are 31.64 (100), 34.32 (002), 36.18 (101), 47.58 (102), 56.52 (110), 62.76 (103), 66.64 (200), 68.84 (201), 69.18 (201), and 77.1. All of the obtained bands approve the hexagonal structure that is consistent with the literature, proving that the powder is extremely crystalline (Hassaan et al. 2020a). Strong, sharp peaks and the absence of bands from other zinc oxide and impurity phases demonstrate the ZnO-NPs’ high purity and crystallinity. The particle sizes of the prepared green ZnO-NPs are determined by the Scherrer formula and are found to be in the range of 2.4–3.6, respectively. The Debye–Scherrer Eq. (3) uses the full width at half maximum (FWHM) of the 101 anatase band to estimate the crystallite sizes of the ZnO-NPs in Table 1 (Hassaan et al. 2020a).

3 $${C}_{S}=\frac{0.89\lambda }{\beta cos\theta }$$

where *Cs* designates the crystallite size, *λ* is the XRD wavelength (1.5406 Å), *θ* is the Braggs’ XRD diffraction angle, and *β* is the FWHM in radians.

**Table 1:** Crystal size (CS) calculation of synthesized green ZnO-NPs

Location	CS of green ZnO-NPs (nm)
31.64°	3.2
34.32°	3.6
**36.18°**	3.3
47.58°	2.9
56.52°	2.8
62.76°	2.4
66.64°	3.0
68.84°	2.4
69.18°	2.5
77.01°	3.1

#### Transmission electron microscope (TEM)

**Fig. 6:**
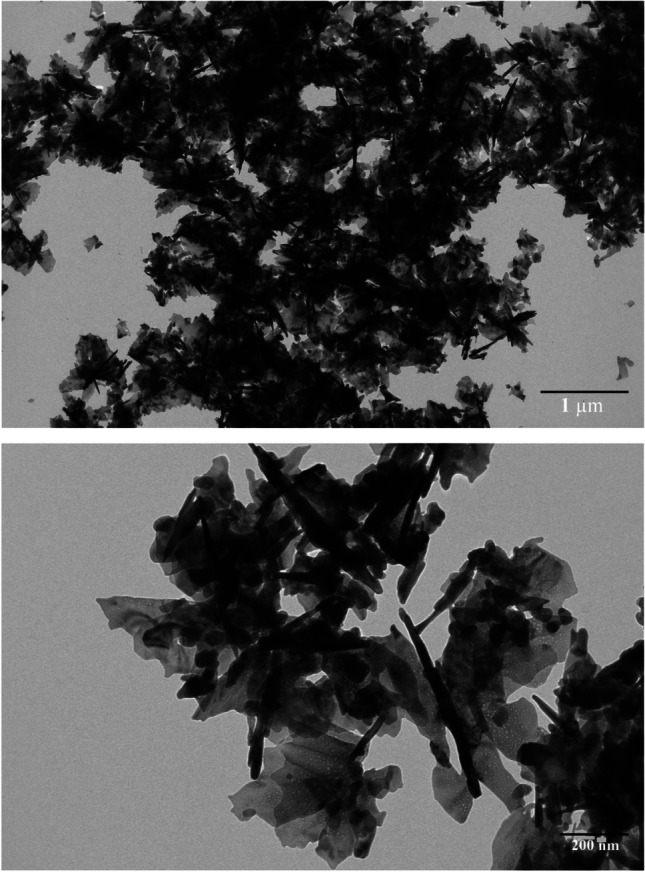
TEM images of synthesized green ZnO-NPs

TEM was used to further investigate the form and dispersion of nanoparticles (TEM). Green ZnO-NPs with diverse nanostructures, such as needle-like and sheets (leaf-like), have been generated via green synthesis Fig. 6. One day following the preparation process, all of the TEM characterizations were completed. The particle distribution was computed after digitizing the numerous TEM images, and the outcomes were discovered to be in agreement with those of the XRD investigation.

#### Thermal analysis (TGA)

**Fig. 7:**
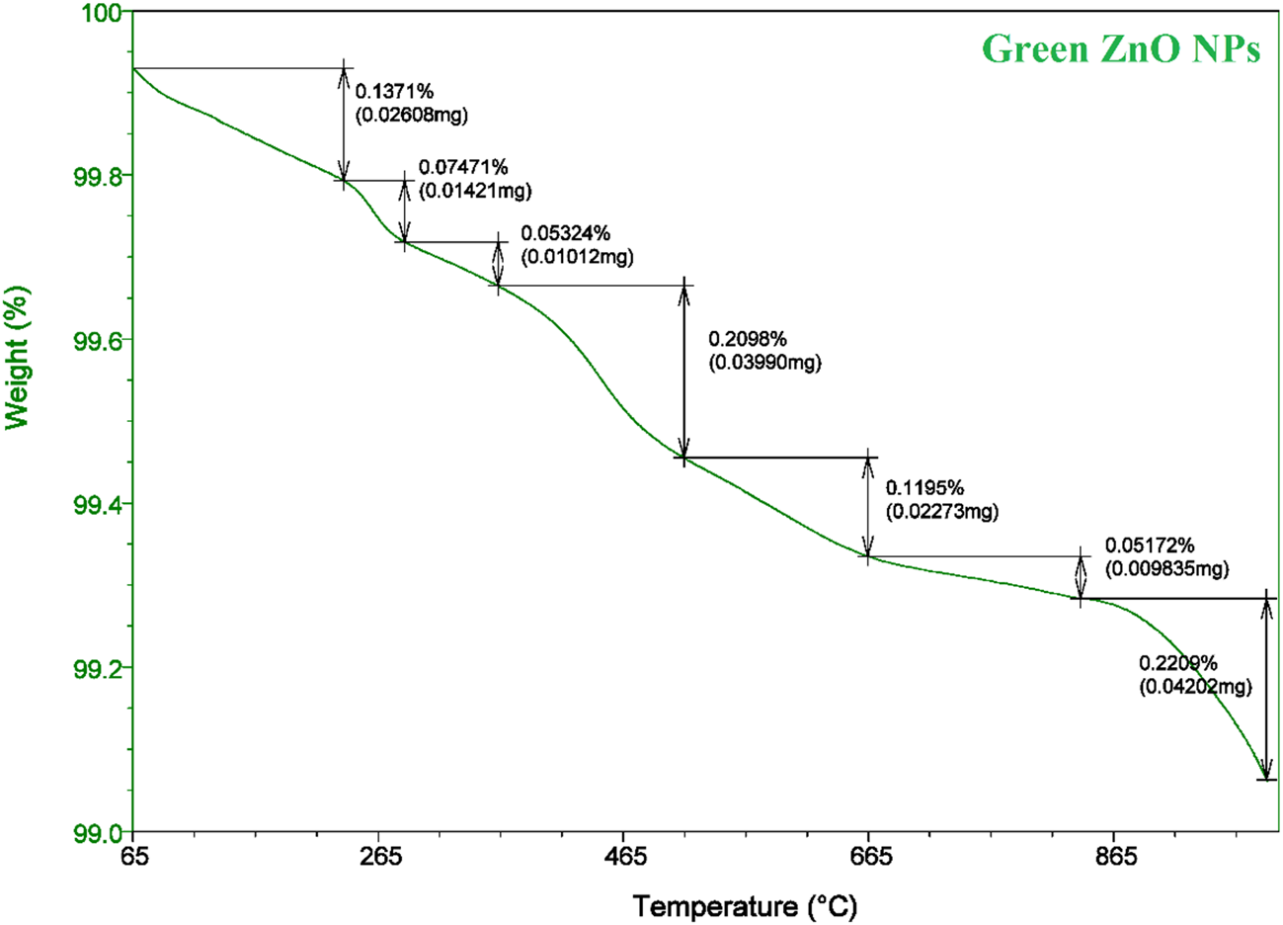
TGA analysis of prepared green ZnO NPs

TGA, the widely accepted method to determine the biomass thermal degradation, is used to investigate the biomass thermal stability (Sanchez-Silva et al. 2012; Carrier et al. 2011). One represents the loss of moisture from the ZnO NPs sample at temperatures between 100 and 210 °C, accounting for 0.137% of its weight, while the remaining six represent the cellulose and lignin degradation at temperatures from 210 to 950 °C, resulting in a weight loss of 0.73% of the green ZnO NPs sample as presented in Fig. 7.

#### BET analysis

**Fig. 8:**
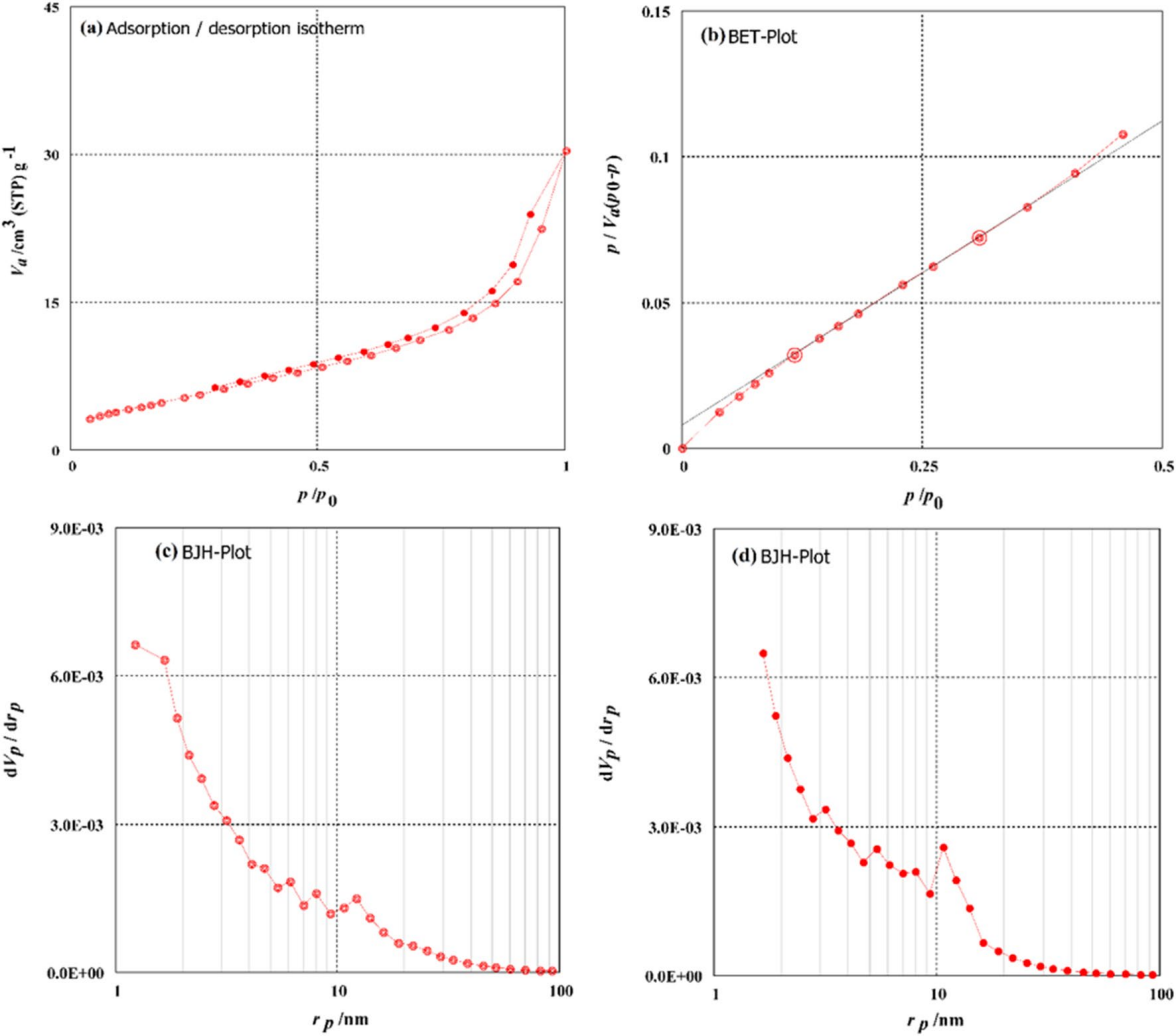
Surface area analysis of synthesized green ZnO NPs (a) N_2_ Adsorption–desorption curve, (b) BET investigation curve, (c) BJH investigation of adsorption curve, (d) BJH analysis of desorption curve

Porous nature of the green ZnO-NPs is investigated using the Brunauer–Emmett–Teller (BET) surface area, as shown in Table 2 (Barrett et al. 1951; Li et al. 2022; Yılmaz et al. 2022). The surface area of ZnO NPs obtained using BET analysis is 20.111 m^2^/g, mono-layer volume (*V*_m_) is 4.6206 cm^3^ (STP) g^−1^, the total volume of pores is 0.04377 cm^3^/g, and the mean diameter of pores (*P*_m_) is 8.7057 nm (Fig. 8). The mesoporous material characteristics are consistent with the average pores having a diameter < 50 nm. Therefore the BJH analysis was applied to the adsorption desorption curve and the achieved data were presented in Table 2.

**Table 2:** Analysis information of synthesized green ZnO-NPs using BET and BJH analysis methods

BET analysis				BJH ads. analysis			BJH des. analysis		
*V*_m_	*S*_BET_	*V*_T_	*P*_m_	*V*_P_	*S*_*BJH*_	*r*_p,peak_	*V*_P_	*S*_*BJH*_	*r*_p,peak_
4.6206	20.111	0.04377	8.7057	0.04362	20.08	1.22	0.4048	13.994	1.66

*V*_m_, volume of mono-layer pores (cm^3^ (STP) g^−1^); *S*_BET_, BET specific surface area (m^2^ g^−1^); *V*_T_, volume of total pores (cm^3^ g^−1^); *P*_m_, mean diameter of pores (nm); *V*_P_, measopore volume (cm^3^ g^−1^); *S*_BJH_, surface area measured by BJH analysis method; *r*_p,peak_, mean pore radius by BJH analysis method

### pH impact on the sorption of DB106 dye

**Fig. 9:**
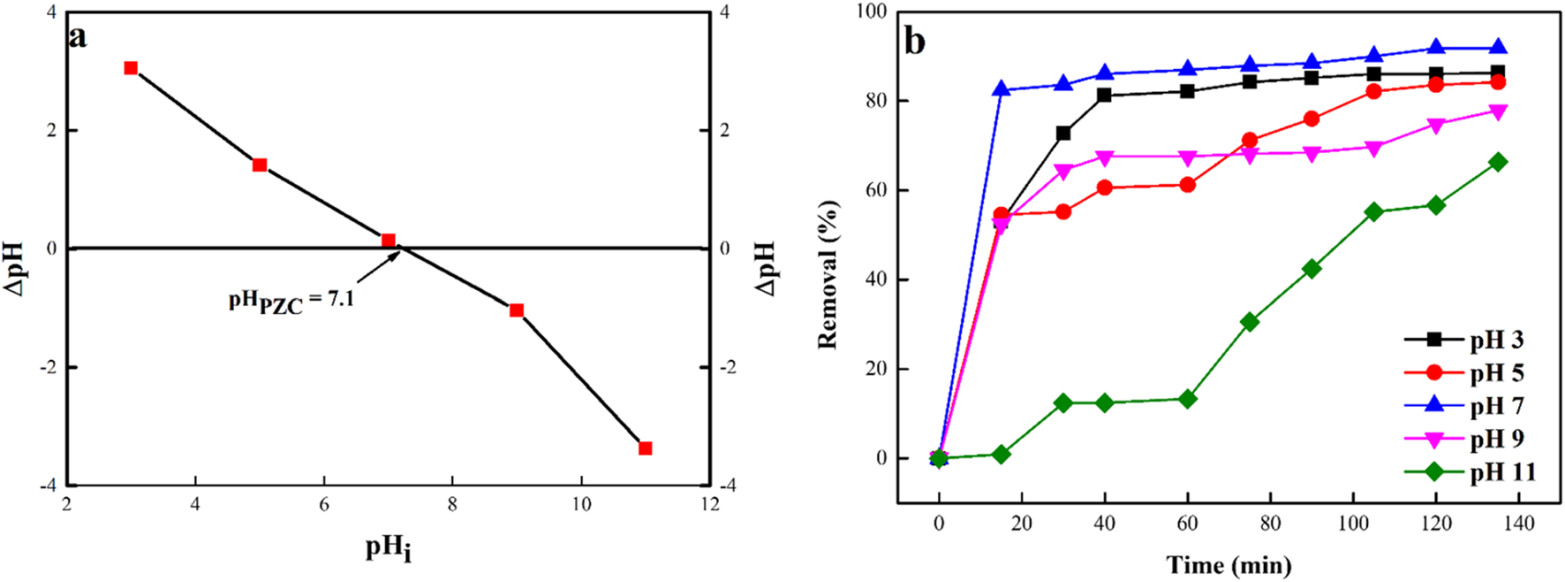
(a) pH_PZC_ of ZnO-NPs and (b) impact of pH of the confiscation of DB106 dye

The sorption technique’s effective treatment of water-soluble solutions is impacted by the sorbent properties, ion selectivity of heavy metals, pH of the solution, temperature, contact time and co-existing ions. It generally varies with the sorbent properties like the size of the particles, pore-size structure, pH_pzc_ and the precise surface area (*S*_BET_) (Wei et al. 2022). A critical regulatory factor in the sorption method is the pH value of the solution. It impacts the degree of solute ionization as well as the sorbent surface charge and the functional groups’ dissociation on the sorbent active sites (Amin 2009; Markandeya et al. 2017). The pH_PZC_ of the sorbent was found to be 7.1, which meant that at pH below the pH_PZC_, the active surface sites on the sorbent were positively charged and while at pH greater than pH_PZC_, the active surface sites on the sorbent were negatively charged (Fig. 9a). The percentage of DB106 dye molecules sequestered as the solution pH was increased from pH 2 to 7, and subsequently decreased with increasing pH values, with the optimal sorption of dye molecules noticed at pH 7 (Fig. 9b). At low pH, the adsorption of the dye molecules to the sorbent was due to the electrostatic attraction between the protonated (H^+^) sites on the sorbent and the anionic dye, hence the increased removal of the molecules of dye at the acidic pH. The active sites on the sorbent get deprotonated (OH^–^) when the pH of the solution rises, which causes an electrostatic attraction between the anionic dye molecules and the negative charge sites on the sorbent. Thereby reduced sorption of the dye molecules observed at the basic pH. In the neutral pH (pH 7), the forces of attraction and repulsion were sidestepped and the optimum sequestration of the dye molecules to the sorbent surface sites was a result of the Van der Waals forces acting as an intermolecular force of attraction between the molecules of DB 106 dye molecules and the sorbent active surface sites (Shabaan et al. 2020). The same phenomenon noticed under the impact of pH on the adsorption DB106 dye molecules in this study was reported also in the studies by Salama et al. (2022) and Hassan et al. (2021), where the best sorption of anionic dye molecules was observed at pH 7.

### Dosage impact on the DB 106 dye sorption

**Fig. 10:**
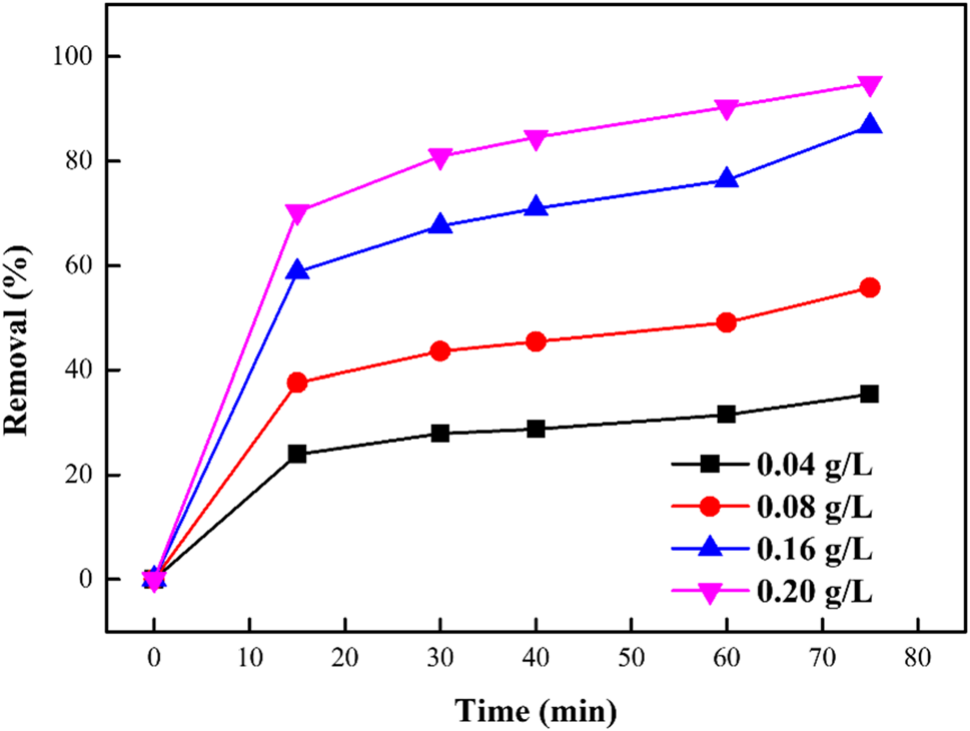
Influence of sorbent dosage of sorption DB106 dye

The sorbent quantity is an extensive parameter in a system that impacts the rate of sorption (Shabaan et al. 2020). As observed from Fig. 10, the % of DB106 dye sequestered from the aqueous solution to the sorbent active surface sites, enhanced with increasing dosage of sorbent used (0.04–0.20 g/L) as time interaction was improved. The maximum sorption of DB106 dye molecules was noticed using the dosage of 0.20 g/L with increasing time of interaction. Enhancing the sorbent dosage led to the offering of more active surface sites on the ZnO-NPs, which improved the adsorption of DB106 dye molecules (Salama et al. 2022; Jaber and Jabbar 2021).

### Impact of starting concentration of DB106 dye

**Fig. 11:**
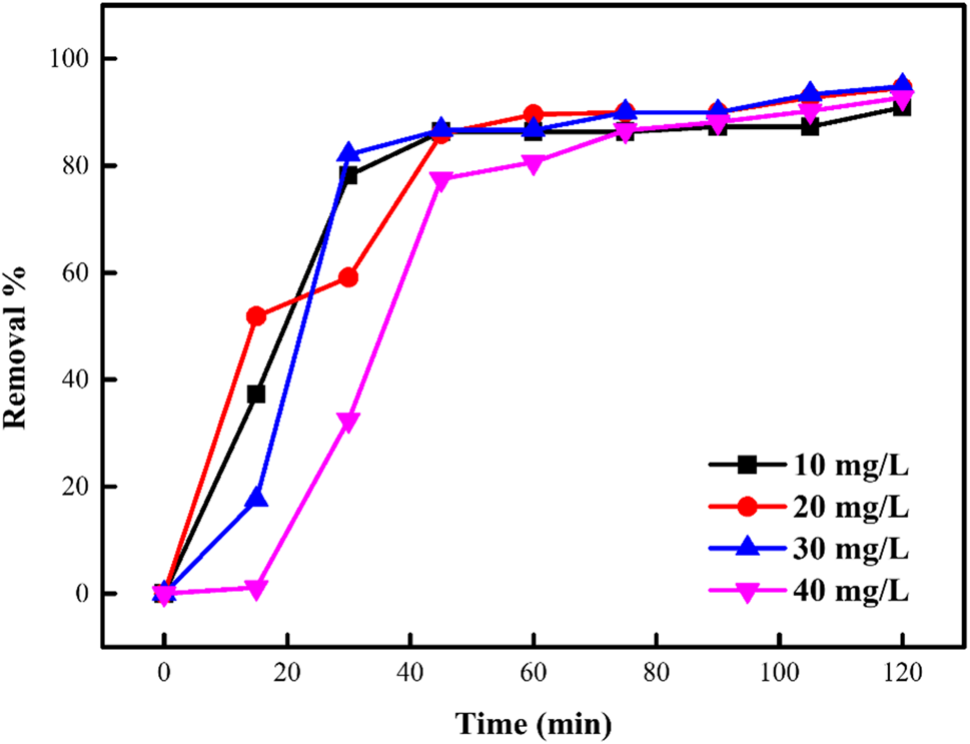
Impact of starting DB106 dye concentration on the confiscation of dye molecules

The impact of beginning concentration of DB106 dye on the sorption process was assessed at DB 106 dye concentrations array of 10–40 mg/L as represented in Fig. 11. It was detected that increasing the DB106 dye concentration from 10 to 40 mg/L over time, the % of DB106 dye removed decreased with increasing concentration of DB106 dye. At low DB106 dye concentrations, the number of active surface sites on the sorbent available was high relative to the DB106 dye concentration. Hence, dye molecules had sufficient active surface sites on the sorbent to interact with and occupy. With improvement in the DB106 dye concentration to 40 mg/L, the quantity of active surface sites on the sorbent available for the dye molecules was reduced, thereby leading to reduced interaction and decreased removal of dye molecules (Jaber and Jabbar 2021). The improvement in the elimination of dye molecules as the beginning dye concentration was increased was due to reduced mass transfer resistance between the water-soluble and solid stages that happens with improving the preliminary DB106 dye concentration (Amin 2009; Nourmoradi et al. 2015).

### Impact of stirring speed on DB 106 dye sorption

**Fig. 12:**
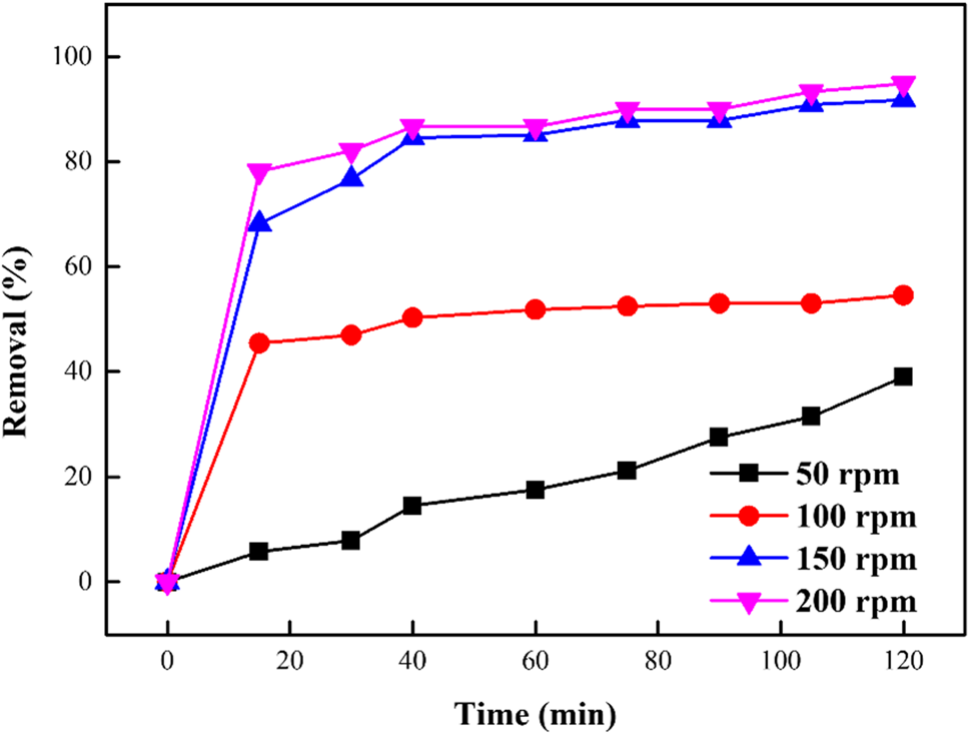
Contact time impact on the DB106 dye adsorption

A key mass transfer factor that impacts the sorption process is the stirring or shaking speed. The impact of the shaking speed on the sequestration of DB106 dye onto the sorbent was assessed at varying shaking speeds of 50–200 rpm. Data reported in Fig. 12 show that the % of DB106 dye confiscated to the sorbent improved with increasing shaking speed as the time of shaking was increased, with optimum removal noticed using the shaking speed of 200 rpm. At low shaking speed, the time that was needed to attain equilibrium was longer and increasing the agitation speed led to an increased diffusion rate of the molecules of dyes from the majority liquid to the liquid boundary layer surrounding the particles which became above average owing to improved instability and reduced the liquid boundary layer thickness as the speed of agitation enhanced (Bhatti et al. 2015).

### Temperature influence on the sorption of DB106 dye

**Fig. 13:**
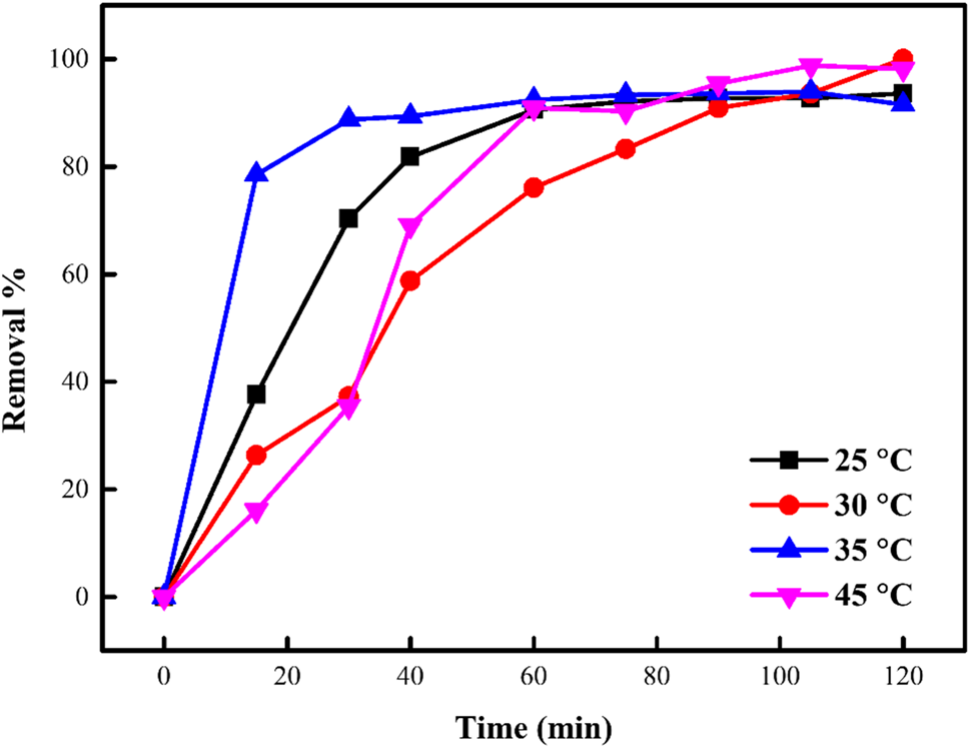
Temperature impact on the confiscation of DB106 dye

It was detected that the influence of temperature on the confiscation of DB106 dye improved with increasing temperatures (Fig. 13). The optimal sorption of the DB106 dye was reported at 45 °C. This was attributed to the dye solubility, which reduces as time and temperature were enhanced and thereby leading to the high rate of sorption of dye molecules to the green ZnO-NPs. Also, due to the increased mobility of the molecules of dyes at elevated temperatures led to the increased sorption of dye (Karthik et al. 2020). As reported in the study of Bhatti and Nausheen (2015), increased removal at elevated temperatures was attributed to the development in the pore numbers on the green ZnO-NPs surface. Also, the green ZnO-NPs outer surface thickness was reported to be decreased and the dye molecules kinetic energy enhanced at elevated temperatures, thereby leading to the DB106 dye molecules being effortlessly sorbed into the sorbent surface.

### Kinetic models

**Fig. 14:**
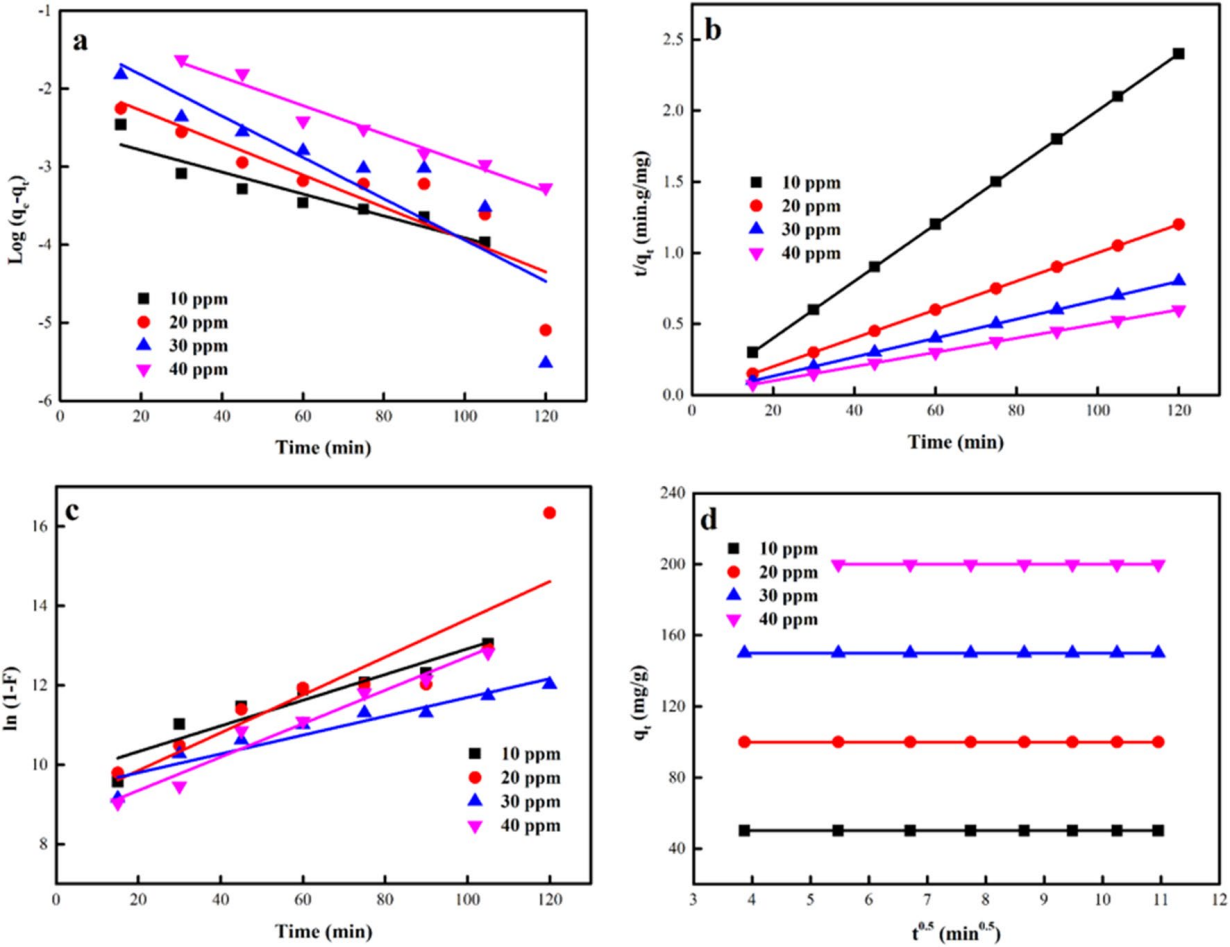
Linear plots of (a) FO, (b) SO, (c) ID and (d) FD models

The adsorption kinetic is utilized to research the variation in the adsorption extent over time and this could present a basis for a process model in engineering and insight into the sorption mechanism (Eldeeb et al. 2022a, b; Aigbe et al. 2022a). To assess the sorption of DB 106 dye to ZnO-NPs, the pseudo-first-order (FO), pseudo-second-order (SO), intraparticle diffusion (ID) and film diffusion (FD) models. The data of the fitting of the linear plot of the different models are represented in Fig. 14. The coefficient of regression (*R*^2^) closeness to unity (1) was used to describe the magnitude of the determined model appropriateness to the data. From the determined parameters in Tables 3, 4, 5, and 6, using 0.2–0.3 g/L of the sorbent, it was found that the *R*^2^ values of the SO kinetic model best depicted the sorption process of DB106 dye to the sorbent in comparison to other functional models. The sorption of DB106 dye to ZnO-NPs was indicative of a chemical sorption process, which elaborates the valency forces allocation or exchange of electrons between the sorbent and sorbate (Eldeeb et al. 2022a, b; Jawad et al. 2022). Also exploring the sorption method of the DB106 dye to ZnO-NPs using ID and FD kinetic models, it was discovered that the fitting lines of these models did not pass via the origin of their various plots (Fig. 14c,d), which showed that the rate-limiting step comprises of various paths such as external diffusion did not control the adsorption of DB106 dye molecules to ZnO-NPs. The values of *R*^2^ were comparatively close to 0.9 using 0.3 g/L of the adsorbent for both models but were moderately low by 0.2 g/L of the ZnO-NPs.

**Table 3:** 0.3 g/L was used to determine the factors of the FO and SO models.

Parameter		FO		SO		
ZnO-NPs	DB106 dye Initial Conc. (mg L^−1^)	*k*_*1*_	*R*^2^	*k*_2_ × 10^3^	*h*	*R*^2^
0.3 g L^−1^	25	3.4545	0.6915	0.35721	6523.2	0.981
	30	2.7636	0.9428	0.09189	1134.4	0.926
	35	0.4606	0.5609	11.7037	16,920.5	0.999
	40	2.9939	0.8085	0.05405	446.7	0.393
	45	0.4606	0.7282	13.4149	4904.4	0.999

**Table 4:** 0.2 g/L was used to calculate the factors of the FO and SO models.

Parameter		FO		SO		
ZnO NPs	DB106 dye Initial Conc. (mg L^−1^)	*k*_1_	*R*^*2*^	*k*_2_ × 10^6^	*h*	*R*^2^
0.2 g L^−1^	25	− 2.51	0.897	0.02	0.0500	1.0
	30	− 1.87	0.790	0.09	0.9000	1.0
	35	− 1.29	0.767	0.01	0.2228	1.0
	40	2.99	0.968	0.01	0.4000	1.0

**Table 5:** The parameters of the ID and FD models were determined at 0.3 g/L

ZnO-NPs	DB106 dye Initial Conc. (mg L^−1^)	ID			FD	
		*K*_*dif*_	*C*	*R*^2^	*K*_*FD*_	*R*^2^
0.3 g L^−1^	25	8.437	31.123	0.9769	0.004	0.8573
	30	6.626	− 9.829	0.9568	0.003	0.9784
	35	0.693	30.605	0.9538	0.000	0.9755
	40	3.679	− 6.756	0.9683	0.003	0.5440
	45	0.383	14.671	0.9822	0.000	0.9653

**Table 6:** The parameters of the ID and FD models were determined at 0.2 g/L

ZnO-NPs	DB106 dye Initial Conc. (mg L^−1^)	ID			FD	
		*K*_*dif*_	*C*	*R*^2^	*K*_*FD*_	*R*^2^
0.2 g L^−1^	10	0.000	49.996	0.6614	0.032	0.8974
	20	0.001	99.992	0.8041	0.048	0.7901
	30	0.002	149.980	0.7123	0.024	0.9066
	40	0.003	199.970	0.8190	0.042	0.9675

### Isotherm model

**Fig. 15:**
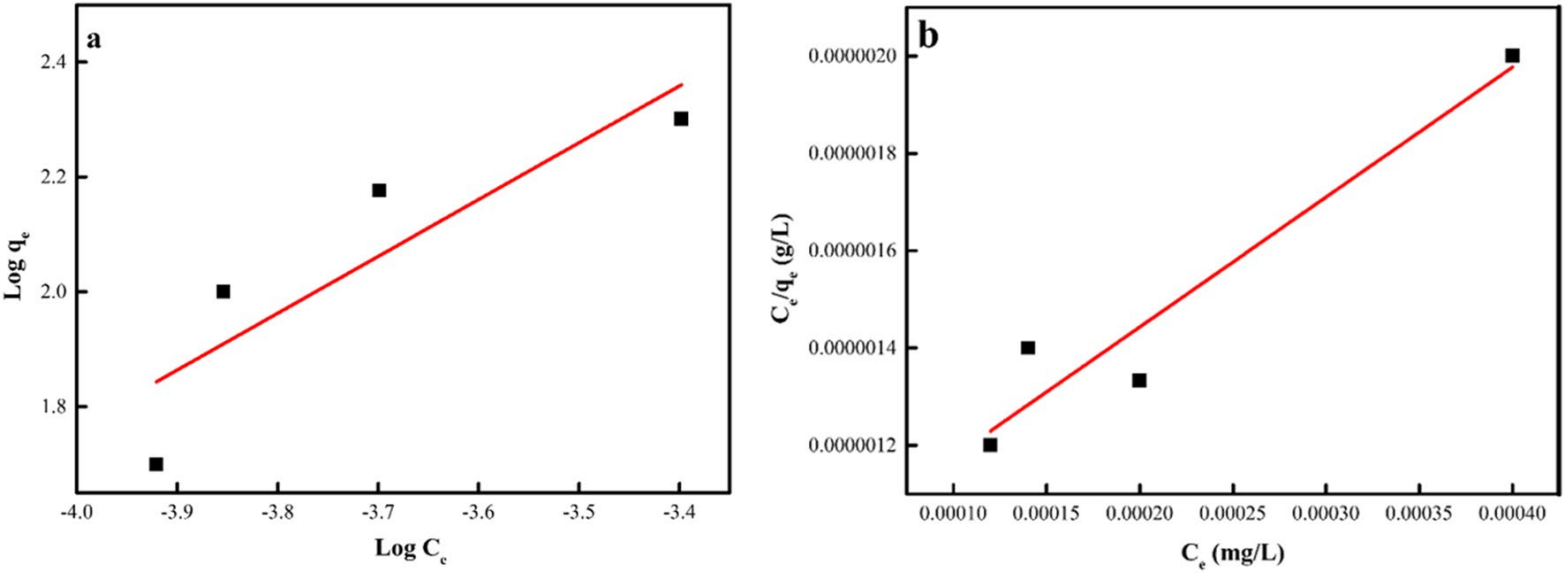
Linear plot (a) LNR and (b) FRH models

To explain the sorption behaviour and get information about the mechanism of sorption, the isotherm models are applied (El Nemr et al. 2010; Aigbe et al. 2020a; Jawad et al. 2022). The Langmuir (LNR) and Freundlich (FRH) models were explored in this study to determine which model ideally described the removal of DB106 dye molecules to ZnO-NPs sorbent. The various plot of these models is shown in Fig. 15. From the determined parameters in Table 7, the LNR was more suitable in describing the sorption of dye molecules to ZnO-NPs due to their determined high *R*^2^ value. The calculated optimum sorption capacity (*Q*_m_) from LNR was 370.37 mg/g. The LNR dimensionless factor *R*_L_ was assessed to be 3.3 $\times {10}^{-5}-8.3\times {10}^{-6}$ using 10–40 mg/L dye concentration and the determined value of *R*_L_ showed a favourable sorption process using ZnO-NPs for DB106 dye sorption. The adsorption process of DB106 dye ions to ZnO-NPs was owing to the monolayer sorption of DB106 dye ions to the consistent surface of the ZnO-NPs.

**Table 7:** The LNR and FRH models determined factors

Isotherm models	Determined isotherm parameters (0.2 g/L)	
LNR	*Q*_*m*_ (mg/g)	370.37
	*K*_*a*_ × 10^3^	3000
	*R*^2^	0.928
FRH	*Q*_*m*_ (mg/g)	280.0
	*K*_*a*_ × 10^3^	529,541.5
	*R*^2^	0.778

### Comparison with other reported work in literatures

According to the comparison of various optimum sorption capacities for DB106 dye removal using different nanomaterials (Table 8), results show that ZnO-NPs can be employed as an effective sorbent for the sequestration of dye molecules from water medium.

**Table 8:** Maximum sorption capacities comparison of various nanomaterials for the sorption of DB106 dye

Sorbents	*Q*_m_ (mg/g)	References
Activated carbons developed from pomegranate peel	42.59, 54.05, and 58.14	(Amin 2009)
Activated carbon from orange peel	107.53	(Khaled et al. 2009)
Oxidized multi-walled carbon nanotubes	500.00	(Sobhanardakani and Zandipak 2015)
ZnO	370.37	This study

## Conclusion and recommendations

The aim of the current study was to reconsider and investigate the potential benefits of utilizing green synthesized ZnO-NPs for DB106 dye removal. The basis of this study was to show how to remove DB106 dye using more widely accessible NPs. Hence, this study reports on the use of green ZnO-NPs adsorbent for DB106 dye adsorption employing the batch adsorption method under varied circumstances in a water-soluble medium. The confiscation of DB106 dye to the green synthesized ZnO-NPs adsorbent was detected to be pH-dependent, with the optimal sorption of DB106 (anionic) dye particles observed at pH 7. DB106 dye adsorption to the ZnO-NPs adsorbent was well-defined by means of the LNR and SO models, with an estimated *Q*_m_ of 370.37 mg/g. The findings of the present study have proved the opportunity and capability of using inexpensive and green synthesis for ZnO-NPs fabrication for the capture of anionic dye particles such as the DB106 dye. Therefore, it is recommended that further research be done to find new, less expensive, and more environmentally friendly adsorbents that can be investigated and used to remove dyes and other harmful substances from the environment. Future studies should also focus on applying smart techniques to develop a broad and precise association for the elimination of industrially hazardous composites such as dyes from the environment.

## Data Availability

The datasets used in this investigation are accessible for review upon request from the corresponding author of the paper.

## References

[CR1] Agrawal A, Sahu K (2006). Kinetic and isotherm studies of cadmium adsorption on manganese nodule residue. J Hazard Mater.

[CR2] Aigbe UO, Onyancha RB, Ukhurebor KE, Obodo KO (2020). Removal of fluoride ions using a polypyrrole magnetic nanocomposite influenced by a rotating magnetic field. RSC Adv.

[CR3] Aigbe UO, Ukhurebor KE, Onyancha RB, Osibote OA, Darmokoeso H, Kusuma HS (2021). Fly ash-based adsorbent for adsorption of heavy metals and dyes from aqueous solution: a review. J MaterRes Technol.

[CR4] Aigbe UO, Ukhurebor KE, Onyancha RB, Ama OM, Osibote OA, Kusuma HS, Okanigbuan PN, Azi SO, Osifo PO (2022a) Dendrimers for environmental remediation. Nanotechnol Environ Remed Chapter 13:243–264. 10.1002/9783527834143.ch13

[CR5] Aigbe UO, Ukhurebor KE, Onyancha RB, Osibote OA, Kusuma HS, Darmokoeso H (2022b) Measuring the velocity profile of spinning particles and its impact on Cr(VI) sequestration. Chem Eng Process 109013. 10.1016/j.cep.2022.109013

[CR6] Al-Arjan WS (2022). Zinc oxide nanoparticles and their application in adsorption of toxic dye from aqueous solution. Polymers.

[CR7] Albo Hay Allah MA, Alshamsi HA (2022) Green synthesis of ZnO NPs using Pontederia crassipes leaf extract: characterization, their adsorption behavior and anti-cancer property. Biomass Convers Biorefin 1–14. 10.1007/s13399-022-03091-y

[CR8] Ali RM, Hassaan MA, Elkatory MR (2020). Towards potential removal of malachite green from wastewater: adsorption process optimization and prediction. Mater Sci Forum.

[CR9] Ali RM, Elkatory MR, Hamad HA (2020). Highly active and stable magnetically recyclable CuFe2O4 as a heterogenous catalyst for efficient conversion of waste frying oil to biodiesel. Fuel.

[CR10] Alim KA, Fonoberov VA, Balandin AA (2005). Origin of the optical phonon frequency shifts in ZnO quantum dots. Appl PhysLett.

[CR11] Amin NK (2009). Removal of direct blue-106 dye from aqueous solution using new activated carbons developed from pomegranate peel: adsorption equilibrium and kinetics. J Hazard Mat.

[CR12] Amirante R, Demastro G, Distaso E, Hassaan MA, Mormando A, Pantaleo AM, Tamburrano P, Tedone L, Clodoveo ML (2018). Effects of ultrasound and green synthesis ZnO nanoparticles on biogas production from Olive Pomace. Energy Proced.

[CR13] Ashkenov N, Mbenkum BN, Bundesmann C, Riede V, Lorenz M, Spemann D, Kaidashev EM, Kasic A, Schubert M, Grundmann M, Wagner G (2003). Infrared dielectric functions and phonon modes of high-quality ZnO films. J Appl Phys.

[CR14] Azari A, Salari M, Dehghani MH, Alimohammadi M, Ghaffari H, Sharafi K, Shariatifar N, Baziar M (2017). Efficiency of magnitized graphene oxide nanoparticles in removal of 2,4-dichlorophenol from aqueous solution. J Mazandaran Univ Med Sci.

[CR15] Azari A, Nabizadeh R, Mahvi AH, Nasseri S (2020). Integrated Fuzzy AHP-TOPSIS for selecting the best color removal process using carbon-based adsorbent materials: multi-criteria decision making vs. systematic review approaches and modeling of textile wastewater treatment in real conditions. Inter J Environ Anal Chem.

[CR16] Azizi S, Mohamad R, Bahadoran A, Bayat S, Rahim RA, Ariff A, Saad WZ (2016). Effect of annealing temperature on antimicrobial and structural properties of bio-synthesized zinc oxide nanoparticles using flower extract of Anchusa italic. Jphotochem Photobiol B Biol.

[CR17] Barrett EP, Joyner LG, Halenda PP (1951). The determination of pore volume and area distributions in porous substances. I. Computations from nitrogen isotherms. J Amer Chem Soc.

[CR18] Bayramoglu G, Arıca MY (2007). Biosorption of benzidine based textile dyes “Direct Blue 1 and Direct Red 128” using native and heat-treated biomass of Trametes versicolor. J Hazard Mater.

[CR19] Bhatti HN, Nausheen S (2015). Equilibrium and kinetic modeling for the removal of Turquoise Blue PG dye from aqueous solution by a low-cost agro waste. Desalin Water Treat.

[CR20] Bhatti HN, Sadaf S, Aleem A (2015). Treatment of textile effluents by low-cost agricultural wastes: batch biosorption study. J Anim Plant Sci.

[CR21] Carrier M, Loppinet-Serani A, Denux D, Lasnier JM, Ham-Pichavant F, Cansell F, Aymonier C (2011). Thermogravimetric analysis as a new method to determine the lignocellulosic composition of biomass. Biomass Bioenerg.

[CR22] Chou TP, Zhang Q, Fryxell GE, Cao G (2007). Hierarchically structured ZnO film for dye-sensitized solar cells with enhanced energy conversion efficiency. Adv Mater.

[CR23] Deng Y, Zhao R (2015). Advanced oxidation processes (AOPs) in wastewater treatment. Curr Pollut Rep.

[CR24] El Nemr A, El-Sikaily A, Khaled A (2010). Modeling of adsorption isotherms of Methylene Blue onto rice husk activated carbon. Egypt J Aquat Res.

[CR25] El Nemr A, Hassaan MA, Elkatory MR, Ragab S, Pantaleo A (2021). Efficiency of Fe3O4 nanoparticles with different pretreatments for enhancing biogas yield of macroalgae Ulva intestinalis Linnaeus. Molecules.

[CR26] Eldeeb TM, Aigbe UO, Ukhurebor KE, Onyancha RB, El-Nemr MA, Hassaan MA, Ragab S, Osibote OA, El Nemr A (2022a) Adsorption of Methylene Blue (MB) dye on ozone, purified and sonicated sawdust biochars. Biomass Convers Biorefin 1–23. 10.1007/s13399-022-03015-w

[CR27] Eldeeb TM, Aigbe UO, Ukhurebor KE, Onyancha RB, El-Nemr MA, Hassaan MA, Osibote OA, Ragab S, Okundaye B, Balogun VA, El Nemr A (2022b) Biosorption of acid brown 14 dye to Mandarin-Biochar-CO-TETA derived from Mandarin peels. Biomass Convers Biorefin 1–21. 10.1007/s13399-022-02664-1

[CR28] Eleryan A, Aigbe UO, Ukhurebor KE, Onyancha RB, Eldeeb TM, El-Nemr MA, Hassaan MA, Ragab S, Osibote OA, Kusuma HS, Darmokoesoemo H, El Nemr A (2022) Copper (II) ion removal by chemically and physically modified sawdust biochar. Biomass Convers Biorefin 1–38. 10.1007/s13399-022-02918-y

[CR29] Elia P, Zach R, Hazan S, Kolusheva S, Porat ZE, Zeiri Y (2014). Green synthesis of gold nanoparticles using plant extracts as reducing agents. Int J Nanomed.

[CR30] El-Nemr MA, Aigbe UO, Ukhurebor KE, Onyancha RB, El Nemr A, Ragab S, Osibote OA, Hassaan MA (2022) Adsorption of Cr^6+^ ion using activated Pisum sativum peels-triethylenetetramine. Environ Sci Pollut Res 29:91036–91060. 10.1007/s11356-022-21957-6 10.1007/s11356-022-21957-6 PMC9722890 35881295

[CR31] Emegha JO, Ukhurebor KE, Aigbe UO, Damisa J, Babalola AV (2022). Synthesis and characterization of Copper Zinc Iron Sulphide (CZFS) thin films. Heliyon.

[CR32] Ghiloufi I, El Ghoul J, Modwi A, El Mir L (2016). Ga-doped ZnO for adsorption of heavy metals from aqueous solution. Mater Sci Semicond Process.

[CR33] Golder AK, Chanda AK, Samanta AN, Ray S (2011). Removal of hexavalent chromium by electrochemical reduction–precipitation: investigation of process performance and reaction stoichiometry. Sep Purif Technol.

[CR34] Guida S, Van Peteghem L, Luqmani B, Sakarika M, McLeod A, McAdam EJ, Jefferson B, Rabaey K, Soares A (2022). Ammonia recovery from brines originating from a municipal wastewater ion exchange process and valorization of recovered nitrogen into microbial protein. Chem Eng J.

[CR35] Harper TR, Kingham NW (1992). Removal of arsenic from wastewater using chemical precipitation methods. Water Environ Res.

[CR36] Hassaan MA, Pantaleo A, Tedone L, Elkatory MR, Ali RM, Nemr AE, Mastro GD (2019). Enhancement of biogas production via green ZnO nanoparticles: experimental results of selected herbaceous crops. Chem Eng Commun.

[CR37] Hassaan MA, Pantaleo A, Santoro F, Elkatory MR, De-Mastro G, Sikaily AE, Ragab S, El Nemr A (2020). Techno-economic analysis of ZnO nanoparticles pretreatments for biogas production from barley straw. Energies.

[CR38] Hassaan MA, El Katory MR, Ali RM, El Nemr A (2020). Photocatalytic degradation of reactive black 5 using photo-Fenton and ZnO nanoparticles under UV irradiation. Egypt J Chem.

[CR39] Hassaan MA, Hosny S, ElKatory MR, Ali RM, Rangreez TA, El Nemr A (2021). Dual action of both green and chemically synthesized zinc oxide nanoparticles: antibacterial activity and removal of Congo red dye. Desalin Water Treat.

[CR40] Hassaan MA, El Nemr A, El-Zahhar AA, Idris AM, Alghamdi MM, Sahlabji T, Said TO (2022). Degradation mechanism of Direct Red 23 dye by advanced oxidation processes: a comparative study. Toxin Rev.

[CR41] Hassan KH, Khammas ZA, Rahman AM (2008). Zinc oxide hydrogen sulfide removal catalyst / preparation, activity test and kinetic study. Al-Khwarizmi Eng J.

[CR42] Hassan SS, El Azab WI, Ali HR, Mansour MS (2015). Green synthesis and characterization of ZnO nanoparticles for photocatalytic degradation of anthracene. Adv Nat Sci Nanosci Nanotechnol.

[CR43] Hassan MJ, Islam M, Akter N, Uddin M, Abir AY, Aoun SB, Hasnat MA (2021). Applicability of gypsum in selective removal of anionic dye molecules from aqueous medium. Appl Water Sci.

[CR44] Jaafari J, Ghozikali MG, Azari A, Delkhosh M, Javid A, Mohammadi AA, Agarwal S, Gupta V, Sillanpää M, Tkachev A, Burakov A (2018). Adsorption of *p*-Cresol on Al_2_O_3_ coated multi-walled carbon nanotubes: response surface methodology and isotherm study. J Ind Eng Chem.

[CR45] Jaber HA, Jabbar MFA (2021). Adsorption of cationic and anionic dyes from aqueous solution using sunflower husk. Chemistry.

[CR46] Jawad AH, Saber SE, Abdulhameed AS, ReghiouaALOthman AZA, Wilson LD (2022). Mesoporous activated carbon from mangosteen (Garcinia mangostana) peels by H3PO4 assisted microwave: optimization, characterization, and adsorption mechanism for methylene blue dye removal. Diam Relat Mater.

[CR47] Jing Z, Zhan J (2008). Fabrication and gas-sensing properties of porous ZnO nanoplates. Adv Mater.

[CR48] Karthik V, Selvakumar P, Sivarajasekar N, Megavarshini P, Brinda N, Kiruthika J, Balasubramani K, Ahamad T, Naushad M (2020) Comparative and equilibrium studies on anionic and cationic dyes removal by nano-alumina-doped catechol formaldehyde composite. J Chem. 10.1155/2020/7617989

[CR49] Khaled A, El Nemr A, El-Sikaily A, Abdelwahab O (2009). Removal of Direct N Blue-106 from artificial textile dye effluent using activated carbon from orange peel: adsorption isotherm and kinetic studies. J Hazard Mater.

[CR50] Kusuma HS, Aigbe UO, Ukhurebor KE, Onyancha RB, Okundaye B, Ama OM, Darmokoesoemo H, Widyaningrum BA, Osibote OA, Balogun VA (2023). Biosorption of methylene blue using clove leaves waste modified with sodium hydroxide. Results in Chemistry.

[CR51] Li S, Zhang Z, Zhang C, He Y, Yi X, Chen Z, Hassaan MA, Nemr AE, Huang M (2022) Novel hydrophilic straw biochar for the adsorption of neonicotinoids: kinetics, thermodynamics, influencing factors, and reuse performance. Environ Sci Pollut Res 1–11. 10.1007/s11356-022-24131-0 10.1007/s11356-022-24131-0 36414889

[CR52] Markandeya S, Shukla SP, Mohan D (2017). Toxicity of disperse dyes and its removal from wastewater using various adsorbents: a review. Res J Environ Toxicol.

[CR53] Naseer M, Aslam U, Khalid B, Chen B (2020). Green route to synthesize Zinc Oxide Nanoparticles using leaf extracts of Cassia fistula and Melia azadarach and their antibacterial potential. Sci Rep.

[CR54] Neolaka YAB, Riwu AAP, Aigbe UO, Ukhurebor KE, Onyancha RB, Darmokoesoemo H, Kusuma HS (2023). Potential of activated carbon from various sources as a low-cost adsorbent to remove heavy metals and synthetic dyes. Results Chem.

[CR55] Nourmoradi H, Ghiasvand AR, Noorimotlagh Z (2015). Removal of methylene blue and acid orange 7 from aqueous solutions by activated carbon coated with zinc oxide (ZnO) nanoparticles: equilibrium, kinetic, and thermodynamic study. Desalin Water Treat.

[CR56] Onyancha RB, Ukhurebor KE, Aigbe UO, Osibote OA, Kusuma HS, Darmokoesoemo H, Balogun VA (2021). A systematic review on the detection and monitoring of toxic gases using carbon nanotube-based biosensors. Sens Bio-Sens Res.

[CR57] Onyancha RB, Aigbe UO, Ukhurebor KE, Kusuma HS, Darmokoesoemo H, Osibote OA, Pal K (2022). Influence of magnetism-mediated potentialities of recyclable adsorbents of heavy metal ions from aqueous solutions - an organized review. Resul Chem.

[CR58] Ragunathan V, Pandurangan J, Ramakrishnan T (2019) Gas chromatography-mass spectrometry analysis of methanol extracts from marine red seaweed Gracilaria corticata. Pharmacogn J 11(3):547–554. 10.5530/pj.2019.11.87

[CR59] Salah H, Elkatory MR, Fattah MA (2021). Novel zinc-polymer complex with antioxidant activity for industrial lubricating oil. Fuel.

[CR60] Salama E, Ghanim M, Hassan HS, Amer WA, Ebeid EZ, El-Shazly AH, Ossman M, Elkady MF (2022). Novel aspartic-based bio-MOF adsorbent for effective anionic dye decontamination from polluted water. RSC Adv.

[CR61] Sanchez-Silva L, López-González D, Villaseñor J, Sánchez P, Valverde JL (2012). Thermogravimetric-mass spectrometric analysis of lignocellulosic and marine biomass pyrolysis. Bioresour Technol.

[CR62] Schwermer CU, Krzeminski P, Wennberg AC, Vogelsang C, Uhl W (2018). Removal of antibiotic resistant E. coli in two Norwegian wastewater treatment plants and by nano-and ultra-filtration processes. Water Sci Technol.

[CR63] Semerjian L, Ayoub G (2003). High-pH–magnesium coagulation–flocculation in wastewater treatment. Adv Environ Res.

[CR64] Shabaan OA, Jahin HS, Mohamed GG (2020). Removal of anionic and cationic dyes from wastewater by adsorption using multiwall carbon nanotubes. Arab J Chem.

[CR65] Shoaib AG, El-Sikaily A, El Nemr A, Mohamed AE, Hassan AA (2022a) Testing the carbonization condition for high surface area preparation of activated carbon followed Type IV from green alga *Ulva lactuca*. Biomass Convers Biorefin 12:3303–3318. 10.1007/s13399-020-00823-w

[CR66] Shoaib AG, El-Sikaily A, El Nemr A, Mohamed AE, Hassan AA (2022b) Preparation and characterization of highly surface area activated carbons followed Type IV from marine red alga (*Pterocladia capillacea*) by zinc chloride activation. Biomass Convers Biorefin 12:2253–2265. 10.1007/s13399-020-00760-8

[CR67] Sobhanardakani S, Zandipak R (2015). Removal of anionic dyes (direct blue 106 and acid green 25) from aqueous solutions using oxidized multi-walled carbon nanotubes. Iran J Health Sci.

[CR68] Soliman EA, Elkatory MR, Hashem AI, Ibrahim HS (2018). Synthesis and performance of maleic anhydride copolymers with alkyl linoleate or tetra-esters as pour point depressants for waxy crude oil. Fuel.

[CR69] Song Z, Williams C, Edyvean R (2004). Treatment of tannery wastewater by chemical coagulation. Desali.

[CR70] Sudarni DHA, Aigbe UO, Ukhurebor KE, Onyancha RB, Kusuma HS, Darmokoesoemo H, Osibote OA, Balogun VA, Widyaningrum BA (2021). Malachite green removal by activated potassium hydroxide clove leaves agro-waste biosorbent: characterization, kinetics, isotherms and thermodynamics studies. Adsorpt Sci Technol.

[CR71] Ukhurebor KE, Aidonojie PA (2021). The influence of climate change on food innovation technology: review on topical developments and legal framework. Agric Food Secur.

[CR72] Ukhurebor KE, Athar H, Adetunji CO, Aigbe UO, Onyancha RB, Abifari O (2021). Environmental implications of petroleum spillages in the Niger Delta Region of Nigeria: a review. J Environ Manage.

[CR73] Ukhurebor KE, Aigbe UO, Onyancha RB, Nwankwo W, Osibote OA, Paumo HK, Ama OM, Adetunji CO, Siloko IU (2021). Effect of hexavalent chromium on the environment and removal techniques: a review. J Environ Managem.

[CR74] Ukhurebor KE, Athar H, Adetunji CO, Aigbe UO, Onyancha RB, Abifarin O (2021). Environmental implications of petroleum spillages in the Niger Delta region of Nigeria: a review. J Environ Managem.

[CR75] Ukhurebor KE, Onyancha RB, Aigbe UO, UK-Eghonghon G, Kerry RG, Kusuma HS, Darmokoesoemo H, Osibote OA, Balogun VA (2022). A methodical review on the applications and potentialities of using nanobiosensors for diseases diagnosis. BioMed Res Int.

[CR76] Ukhurebor KE, Aigbe UO, Onyancha RB, UK-Eghonghon G, Balogun VA, Egielewa PE, Ngonso BF, Imoisi SE, Ndunagu JN, Kusuma HS, Darmokoesoemo H (2022a) Greenhouse gases emission: perception during the COVID-19 pandemic. BioMed Res Int. 10.1155/2022/6166276 10.1155/2022/6166276 PMC9553500 36246992

[CR77] Wang X, Cai W, Lin Y, Wang G, Liang C (2010). Mass production of micro/nanostructured porous ZnO plates and their strong structurally enhanced and selective adsorption performance for environmental remediation. J Mater Chem.

[CR78] Wei Y, Wang L, Li H, Yan W, Feng J (2022) Synergistic fluoride adsorption by composite adsorbents synthesized from different types of materials—a review. Front Chem 10:900660. 10.3389/fchem.2022.900660 10.3389/fchem.2022.900660 PMC9114667 35601557

[CR79] Xu CX, Sun XW, Dong ZL, Cui YP, Wang BP (2007). Nanostructured singlecrystalline twin disks of zinc oxide. Cryst Growth Des.

[CR80] Yang J, Shojaei S, Shojaei S (2022). Removal of drug and dye from aqueous solutions by graphene oxide: adsorption studies and chemometrics methods. NPJ Clean Water.

[CR81] Yılmaz M, Eldeeb TM, Hassaan MA, El-Nemr MA, Ragab S, El Nemr A (2022). The use of Mandarin-Biochar-O3-TETA (MBT) produced from Mandarin peels as a natural adsorbent for the removal of Acid Red 35 (AR35) dye from water. Environ Process.

[CR82] Yu JG, Yu LY, Yang H, Liu Q, Chen XH, Jiang XY, Chen XQ, Jiao FP (2015). Graphene nanosheets as novel adsorbents in adsorption, preconcentration and removal of gases, organic compounds and metal ions. Sci Total Environ.

[CR83] Zazouli MA, Azari A, Dehghan S, Malekkolae RS (2016). Adsorption of methylene blue from aqueous solution onto activated carbons developed from eucalyptus bark and *Crataegus*
*oxyacantha* core. Water Sci Technol.

[CR84] Zeng H, Cai W, Liu P, Xu X, Zhou H, Klingshirn C, Kalt H (2008). ZnO-based hollow nanoparticles by selective etching: elimination and reconstruction of metal-semiconductor interface, improvement of blue emission and photocatalysis. ACS Nano.

